# Beyond Discrimination: Generative AI Applications and Ethical Challenges in Forensic Psychiatry

**DOI:** 10.3389/fpsyt.2024.1346059

**Published:** 2024-03-08

**Authors:** Leda Tortora

**Affiliations:** School of Nursing and Midwifery, Trinity College Dublin, Dublin, Ireland

**Keywords:** forensic psychiatry, forensic AI, generative AI, generative artificial intelligence, discriminative AI, ethical AI, large language models, large generative AI models

## Abstract

The advent and growing popularity of generative artificial intelligence (GenAI) holds the potential to revolutionise AI applications in forensic psychiatry and criminal justice, which traditionally relied on discriminative AI algorithms. Generative AI models mark a significant shift from the previously prevailing paradigm through their ability to generate seemingly new realistic data and analyse and integrate a vast amount of unstructured content from different data formats. This potential extends beyond reshaping conventional practices, like risk assessment, diagnostic support, and treatment and rehabilitation plans, to creating new opportunities in previously underexplored areas, such as training and education. This paper examines the transformative impact of generative artificial intelligence on AI applications in forensic psychiatry and criminal justice. First, it introduces generative AI and its prevalent models. Following this, it reviews the current applications of discriminative AI in forensic psychiatry. Subsequently, it presents a thorough exploration of the potential of generative AI to transform established practices and introduce novel applications through multimodal generative models, data generation and data augmentation. Finally, it provides a comprehensive overview of ethical and legal issues associated with deploying generative AI models, focusing on their impact on individuals as well as their broader societal implications. In conclusion, this paper aims to contribute to the ongoing discourse concerning the dynamic challenges of generative AI applications in forensic contexts, highlighting potential opportunities, risks, and challenges. It advocates for interdisciplinary collaboration and emphasises the necessity for thorough, responsible evaluations of generative AI models before widespread adoption into domains where decisions with substantial life-altering consequences are routinely made.

## 1 Introduction: discriminative vs generative AI

Generative Artificial Intelligence (GenAI) is a subfield of artificial intelligence which uses machine learning and deep learning techniques to generate ‘seemingly new’ human-like content, such as text, images, audio, and video, in response to various prompts, which are specific instructions provided to the AI system to execute a particular task or achieve a specific outcome.

Unlike the previously prevalent paradigm, known as discriminative artificial intelligence, which primarily focuses on discrimination tasks, such as classifying or differentiating between classes in a given dataset, generative AI models distinguish themselves by their capacity to both discriminate and generate new information based on the input data (1).

Discriminative AI models, mainly used for supervised machine-learning tasks like classification or regression, are algorithms designed to classify data instances by learning the decision boundaries that separate different classes or labels within a dataset. Examples of discriminative models include Support vector machines (SVMs), Decision Trees, Random Forests and Logistic Regression. On the other hand, generative AI models, mostly used in unsupervised and semi-supervised machine learning tasks like clustering and dimensionality reduction, are statistical models that learn regularities and patterns within input data and then use this acquired knowledge to generate novel data instances that share similarities with the original training data. Common examples of generative models include Generative Adversarial Networks (GANs), Hidden Markov models, Bayesian Network Autoregressive models and Latent Dirichlet Allocation (LDA) (2).

From a mathematical perspective, a discriminative machine learning approach trains a model by optimising parameters to maximise the conditional probability P(Y|X). In contrast, a generative model learns parameters by maximising the joint probability P(X, Y), relying on Bayes’ Theorem (3). Consequently, unlike discriminative algorithms that focus on discerning decision boundaries, generative models produce artefacts with a wide range of variety and complexity (4). Additionally, while discriminative models aims for deterministic outcomes, the outputs of generative models are probabilistic and exhibit intrinsic variability (5).

The development of powerful generative AI models was prompted by the introduction of the Transformer neural network architecture in 2017 (6), which marked a significant milestone in machine learning research. Moreover, recent years have witnessed a surge in popularity and a growing interest in the application of generative models, especially since the release of ChatGPT, the popular conversational chatbot launched by OpenAI in November 2022 (7), which brought the concept of generative AI to the general public.

ChatGPT is an example of large language models (LLMs), which are deep learning models programmed to understand and generate natural language; these models, having been trained on a massive corpus of textual data, are able to produce human-like text and perform a range of language- related-tasks (i.e. text generation, question answering, language translation and more), interacting with the user conversationally (8, 9).

Nevertheless, it is important to note that generative AI can generate a wide array of outputs beyond text. For this reason, throughout this paper, the broader term ‘large generative AI models (LGAIMs)’ will be adopted to encompass all the different types of generative AI models, of which large language models (LLMs) are only a subset (10).

## 2 Types of large generative AI models

Large generative AI models (LGAIMs) comprise several subsets of generative AI models designed to generate realistic content across different modalities, such as large language models (LLMs), producing text (e.g., GPT-4, ChatGPT, Bard, Bing) and unimodal and multimodal models generating other media, such as images (e.g., Stable Diffusion, DALL·E 3, Bing Image Creator), videos (e.g., Synthesia, Imagen Video), audio (e.g., MusicLM, Musenet) and more.

Large generative AI models comprise several billion parameters, are trained on large datasets, and rely on significant computational resources. Many large generative AI models are currently in use, and their numbers continue to grow as AI experts experiment with existing models. LGAIMs can be classified according to several criteria, one of which is to categorise them by their underlying architecture. Generative AI comprises a variety of models employing different training mechanisms and output generation processes. At present, the most prevalent generative AI models are:

### 2.1 Generative adversarial networks

GANs are a class of models introduced in 2014 by Ian (11) A Generative Adversarial Network (GAN) consists of two neural networks: a generative model, known as the Generator (G), and a discriminative model, known as the Discriminator (D), working jointly in an adversarial manner to generate realistic data (12, 13).

GANs are best suited for tasks requiring the creation of authentic-looking data, such as images (14) and videos (15), favouring their use in industries such as entertainment and advertising, but also exposing them for potential malicious uses, such as deepfakes generation (16).

### 2.2 Transformer-based models

Also called ‘foundation models’ (17) because they serve as the foundation upon which many other AI models are built, Transformers were introduced in 2017 (6) by a team of Google researchers.

A Transformer model is a type of neural network relying on a set of mathematical techniques called attention mechanisms or self-attention mechanisms; these mechanisms assign weights to each input representation and dynamically learn the most relevant information from the input data. The resulting output is obtained by computing a weighted sum of the input values, determining the weights through a compatibility function relating the query with its corresponding key (6, 18).

Those features allow transformer models to learn context by capturing relationships within sequential data, like the words in a sentence, making them ideal for tasks like text generation, and content and code completion. As a result, they have been highly successful in natural language processing (NLP) applications, being the foundation upon which the most popular type of generative AI models, large language models (LLMs), are built. Common subsets of Transformer-based models include Bidirectional Transformers (BERT) Models (19) and Generative Pre-Trained Transformers (GPTs) (20), such as GPT-4, GPT-3, T5 (Text-To-Text Transfer Transformer) and more.

### 2.3 Diffusion models

Diffusion models were developed by Stanford researchers in 2015 (21). They are probabilistic generative models that work by iteratively injecting Gaussian noise into the data. Then, a series of probabilistic denoising steps are applied to reverse this procedure and generate new data samples (22).

Diffusion models have found applications, especially in image generation (23), synthesis (24), and image super-resolution (25). They are the architecture of popular image generation services, such as Dall-E 2, Stable Diffusion, and Midjourney. In addition, they showed promising results in text-to-speech (26), text-to-video (27) and text-to-3D (28).

### 2.4 Variational autoencoders

Variational Autoencoders (VAEs) were introduced in 2013 by Kingma & Welling (29); they are generative models that encode input data into a lower-dimensional latent space and subsequently reconstruct it to its original form. This process involves three components: an Encoder, compressing input data into a probabilistic latent space, the Latent Space, retaining the compressed knowledge, and a Decoder, reconstructing the input data from the compressed latent space (30).

VAEs have found wide applications in several tasks, including image (31), text (32) and music generation (33). Furthermore, VAEs also excel at data compression (34), anomaly detection (35) and missing data imputation (36), and carry the potential for innovation in areas such as finance, speech/audio source separation, and bio signal applications (30).

### 2.5 Neural radiance fields

Neural Radiance Field (NeRF) is a novel approach in computer graphics and computer vision introduced by Mildenhall et al. in 2020 (37).

NeRFs are novel view synthesis methods, mainly applied to create highly detailed and photo-realistic 3D reconstructions of scenes based on 2D images; they achieve this through volume rendering techniques and implicit neural scene representations, often employing multi-layer perceptrons (MLPs) to synthesise novel views of 3D scenes by learning both their geometry and lighting characteristics (38). Therefore, NeRF models have found diverse applications across fields such as photo-realistic 3D editing (39), medical 3D image reconstruction (40), and neural scene representations for world mapping (41). NeRFs have also shown potential in areas like the industry and robotics domain (42), autonomous navigation (43), and augmented and virtual reality (44), where they carry the potential to lead to more efficient techniques for capturing and generating human 3D avatars and objects in the metaverse (45, 46).

Finally, large generative AI models (LGAIMs) can be broadly categorised into two main types: unimodal models and multimodal models. Unimodal models are designed to process just one type of input and generate content based on prompts from the same data format; examples of unimodal models are OpenAI’s GPT-3, NVIDIA’s StyleGAN2 or Google’s BERT. On the other hand, multimodal models are designed to accept inputs and prompts from different modalities and generate content that combines information from different sources and data formats, such as text and images, resulting in more comprehensive outputs (47); examples of multimodal LGAIMs are OpenAI’s GPT-4, ImageBind by Meta AI, and PaLM 2 by Google.

## 3 Discriminative AI’s applications in forensic psychiatry

Before the recent progress and growing popularity of generative AI, discriminative AI was the dominant paradigm in artificial intelligence applications. In forensic psychiatry and criminal justice, discriminative models were developed to assist forensic psychiatrists and legal professionals in assessment and decision-making processes, for instance, informing decisions about pretrial risk assessment, sentencing, bail, parole, probation, allocation to rehabilitation programmes, timing and discharge conditions, and the need for further evaluations.

The most popular and debated application of AI in forensic psychiatry is violence and recidivism risk assessment. Discriminative AI models have been developed to evaluate and predict the likelihood of violence, recidivism, or other unlawful or harmful outcomes in individuals with a psychiatric or criminal history. Within risk assessment, discriminative algorithms feature many applications, such as predicting the risk of general, violent and sexual recidivism (48–52), forecasting future offences (53, 54) and evaluating risk of violence and aggression in psychiatric settings (55, 56), especially amongst individuals labelled as having an enhanced risk of engaging in violent conducts, such as patients diagnosed with schizophrenia (57–60).

These models classify individuals into different risk levels by analysing a vast range of data, including clinical assessments, patient history, demographic factors, and clinical notes. Additionally, they can incorporate personalised data derived from physiological metrics, such as movement sensors and electronic health records (61).

In recent years, there has been a growing interest in integrating genetic, electrophysiological, and neuroimaging data into algorithmic risk assessment models in psychiatry (62). For instance, AI has been coupled with neuroimaging in a technique defined as ‘AI Neuroprediction’, which is the use of structural or functional brain variables coupled with machine learning techniques to identify neurocognitive markers for the prediction of recidivism (63).

In addition to risk assessment, discriminative AI tools have also been applied to improve diagnostic support, aiming to enhance clinical decision-making and diagnostic accuracy. Discriminative algorithms can analyse various types of data, such as behavioural patterns, speech, and textual data, like patient interviews or questionnaires, through several techniques, like natural language processing (NLP), to acquire diagnostic insights; for instance, they can perform machine-learning-based sentiment analyses to examine the patient’s psychological condition and identify potential risks for harmful behaviours, such as risk factors associated to suicide in youth (64). AI-based decision support system (AI-based DSSs) have been applied to various tasks, from the prediction of mental health disorders (65) to risk assessment and management in patients discharged from medium secure services (MSS) (66).

The aforementioned capabilities also find application in personalised treatment planning; by examining patient histories, symptoms, physiological data and responses to previous treatments, discriminative AI algorithms can provide treatment recommendations, uncovering previously unnoticed targets for intervention and aiding in developing more individualised rehabilitation programs for individuals transitioning the criminal justice system. Furthermore, discriminative models, by predicting the potential treatment’s effectiveness for each individual, could help optimise resource allocation. This issue is particularly relevant in forensic psychiatry, where institutions often grapple with acquiring sufficient resources to meet patients’ needs and specialised service demands due to limited staff and financial support.

Nonetheless, it is crucial to highlight that the applications of AI in forensic psychiatry raise several legal and ethical issues that, since their advent, have largely yet to be addressed. While technologies develop at an incredibly fast pace, regulatory policies about their applications struggle to keep up.

The outputs of AI forensic risk assessment tools, relying on datasets reflecting historical biases and ongoing prejudice, have been shown to discriminate against historically marginalised groups in society, perpetuating and amplifying societal systems of inequality. For instance, AI forensic risk assessment algorithms exhibit racial and gender bias, as they systematically overclassify Black defendants and women in higher-risk groups for criminal recidivism (67) and several issues have been raised about these models’ lack of fairness, accuracy and transparency (68). Furthermore, AI-based decision support systems (DSSs), perpetuating biased decision-making, lead to harmful and discriminating outcomes, such as unfair allocation of resources (69), and the increased use of predictive algorithms by law enforcement for predictive policing results in increased surveillance of marginalised groups, raising concerns about privacy and civil liberties (70).

Thus, while it is evident that the criminal justice system continues to face challenges related to the implementation of emerging technologies, we are now entering a new era of AI, marked by the advent of generative artificial intelligence, which is expected to exacerbate them further.

## 4 Generative AI’s transformative impact on forensic psychiatry

The recent advancements in generative AI and the continuous evolution of large generative AI models (LGAIMs) impact multiple societal sectors, from business and healthcare to education and science. Their influence is further extending to critical areas like courtrooms, correctional facilities, and psychiatric settings, where generative AI models hold the potential to reshape forensic mental health practices and law enforcement procedures. In this paragraph, it will be explored how generative models, through their ability to analyse unstructured data across different formats (multimodal generative AI) and generate new synthetic realistic data (data generation and data augmentation), carry the promise not only to influence traditional discriminative AI applications, like risk assessment and personalised treatment design but also to create new opportunities in areas previously underexplored, such as training and education.

Multimodal generative AI models refer to a type of artificial intelligence model designed to process and integrate a vast amount of different data types, for instance, audio recordings of patient interviews, behavioural video observations, and textual reports from psychiatric assessments, but also neuroimaging, genomic data and electronic health records.

In psychiatry, multimodal generative AI models have shown promising results through their ability to analyse multidimensional health data, aiding to predict treatment trajectories (71), improving data interpretation and assisting in the production of clinical reports (72).

The application of multimodal GenAI models in forensic psychiatry could aid in performing advanced behavioural analyses, thereby facilitating a more comprehensive assessment of the patient’s condition and improving the predictive power of risk assessment tools. By their ability to incorporate the temporal information in the learning process, thus capturing the dynamic evolution of the extracted features for each patient (73), these models can integrate a wide range of contextual information, from verbal to non-verbal cues like tone, facial expressions, and body language, thus enabling the implementation of multimodal sentiment analysis and emotion detection tools, aiming to uncover individuals’ emotional states and predict emotional categories. This enhanced emotion detection capacity could serve the development of advanced multimodal decision support systems (DSSs), providing diagnostic insights and highlighting potential risk factors associated, for instance, with violence, aggression, or self-harm. Moreover, the ability of multimodal generative AI models to detect sudden changes and inconsistencies in emotional states could function as an ‘early warning system’, alerting mental health professionals about concerning patterns of behaviours and triggering further assessment. Finally, multimodal models could help tailor personalised interventions in treatment design and planning by integrating several data sources about a patient’s profile and history.

It is important to note that this capability of enhanced behavioural and emotional analyses might also be misused for concerning applications, for instance, to build lie-detection tools to evaluate the credibility of offenders and witnesses or by attempting to reconstruct a person’s mental state and memories during a specific crime. At the same time, the capacity to analyse and integrate a vast amount of personal data over time, from health records to communication history and social media posts, could also contribute to problematic AI profiling techniques.

In addition to multimodal models, generative AI could further influence forensic psychiatry practices by employing data generation and data augmentation techniques, referring to the ability to synthesise new data samples that share similarities with a given dataset.

The potential of generative AI to generate new data instances can impact treatment design and planning by facilitating the creation of personalised treatment simulations. These simulations involve AI-generated scenarios resembling patient profiles and treatment trajectories, enabling forensic clinicians and professionals to virtually test different treatment approaches before implementation and provide insights into their effectiveness. These simulations hold particular promise in addressing complex cases where the efficacy of treatment is uncertain, helping to optimise resource allocation and to evaluate new policies and interventions for individuals transitioning the criminal justice system. For instance, generative AI models could enhance the development of Digital Twins (DTs), virtual models simulating clinical patient trajectories and treatment effects (74), with the potential to assist in tailoring treatment plans, accelerate drug discovery and improve the efficiency of clinical trials (75).

A newly envisioned application leveraging generative AI's scenario simulation capabilities extends to the often overlooked dimension of training and education, where realistic synthetic scenarios simulating various forensic psychiatric case studies and patient interactions could allow forensic mental health professionals to practice decision-making and assessment skills.

By employing GenAI-powered virtual simulations, current and prospective forensic psychiatrists could practice and refine their diagnostic skills in controlled environments, where interactions with AI-generated patients simulating different psychiatric conditions could enable them to gain insights into different behavioural patterns and identify critical risk factors. These simulations could extend to various environments, such as virtual courtrooms and psychiatric settings, where GenAI-created scenarios could simulate ethical dilemmas to help forensic psychiatrists test and navigate ethically challenging situations they might encounter in practice. In the legal realm, they could also assist in defence by generating counterfactual scenarios to explore how patient outcomes might have unfolded under different circumstances.

Alongside AI-powered simulations, generative AI can employ data augmentation methods, commonly used to expand existing datasets by creating variations of the original data samples. Generative data augmentation techniques have been used to address data scarcity by generating synthetic data to train more robust predictive models for medical diagnosis of multiple mental health conditions (76, 77). By generating synthetic patient profiles, data augmentation tools provide supplementary data for analysis, expanding the training dataset for predictive models facing challenges related to insufficient or unbalanced data. This is an issue notably prevalent in forensic psychiatry, where datasets are often limited due to the sensitive nature of information, potentially resulting in an unbalanced representation of various mental health conditions or behavioural patterns.

Finally, it is crucial to highlight the role of generative AI models as decision-making support tools, considering the increasing number of experts who are consulting these models, especially large language Models (LLMs), looking for guidance on a variety of tasks, such as reviewing mental health evaluations in criminal cases, communicating findings in court, and accessing relevant case studies. The increasing use of generative AI will substantially influence decision-making processes within courtrooms and forensic psychiatry settings, regulating which information is accessed and used for report completion and evaluations, as well as affect the data collection processes and diagnostic assessments, for instance, through recommendations to administer relevant tests, questionnaires or interview questions.

In conclusion, the influence of generative AI on forensic psychiatry extends far beyond its discriminative AI applications, with considerable forthcoming developments and its unique set of possibilities and challenges.

## 5 Differences between discriminative and generative AI applications in forensic psychiatry

Generative and discriminative AI both hold potential for applications in forensic psychiatry, but they differ in how they approach the task in several ways.

First, they have different purposes. The primary goal of generative models is to generate new data instances resembling the training data by modelling the joint probability distribution of the observed data. On the other hand, discriminative models aim to distinguish between different classes or categories in the dataset by learning the conditional probability distribution. Therefore, they produce different outputs; while generative models produce data samples drawn from the learned probability distribution, such as realistic synthetic audio, video or textual content, discriminative models directly output class labels or continuous values, making them suited for different tasks.

Accordingly, discriminative and generative AI have different applications and use cases in forensic psychiatry (Prediction vs Generation). Discriminative models are primarily used for predictions, classifications, and regression tasks (Prediction) and find application in tasks such as violence and recidivism risk assessment, diagnostic support and treatment recommendations. On the other hand, generative models are best suited for tasks requiring data generation, data augmentation and probabilistic modelling (Generation). Generative models not only enhance the effectiveness of traditionally discriminative tasks, such as enabling comprehensive behavioural analyses through multimodal models, but also unlock novel opportunities, for instance, through the development of GenAI-powered simulations tailored for personalised treatments and interventions as well as training and educational purposes.

Generative and discriminative AI models further differ regarding training data requirements; specifically, generative models employ unsupervised learning techniques and are trained on unlabelled data, while discriminative models excel in supervised learning and are trained on a labelled dataset. Consequently, generative AI models require more extensive training data compared to discriminative AI algorithms, which can often perform relatively well with smaller datasets, especially when implementing methods like transfer learning or fine-tuning pre-trained models. Another difference pertains to interpretability; while achieving interpretability is already challenging in discriminative models, it becomes even more intricate with generative ones. Discriminative models, employing labelled data, provide outputs that can be interpreted as class probabilities, providing insights into predictive feature contributions. In contrast, generative models introduce a higher level of complexity, as their outputs may not correspond directly to known classes or categories.

In conclusion, the choice between these approaches will depend on the specific task’s objectives, the desired outcome and the available data. Additionally, a hybrid approach combining both methods could offer benefits from both perspectives, contributing to more comprehensive results.

Finally, it is crucial to emphasise that the applications of discriminative and generative AI in forensic psychiatry must be approached carefully and require thorough analysis and regulation before widespread adoption.

## 6 Ethical and legal challenges of generative AI applications in forensic psychiatry and criminal justice

As previously discussed, large generative AI models (LGAIMs) are rapidly transforming many aspects of modern life, including how we communicate, create, and work, impacting various sectors of society. Nevertheless, generative AI models, like other transformative technologies, while harbouring enormous potential, also carry significant risks, and their application raises several ethical and legal concerns.

Misuses of this technology, especially in the fields of forensic psychiatry and criminal justice, might result in significant harm spanning from discrimination to predictive policing, mass surveillance and profiling, impacting individuals’ freedom, right to a fair process, allocation of resources and education of the future generation of legal and mental health professionals. This paragraph will present an overview of some of the pivotal challenges associated with generative AI applications in forensic psychiatry and criminal justice.

The discussion will begin by examining the impact of generative AI on some of the prevalent challenges in AI implementation in forensic psychiatry and criminal justice, encompassing issues such as biases and criminalisation, lack of transparency and interpretability, data privacy, and autonomy. Subsequently, the discourse will delve into GenAI-specific challenges, covering topics such as hallucinations, deepfake fabrications, and homogenisation, along with issues like overreliance. Finally, the analysis will address broader societal concerns about the implementation of generative AI in society, such as environmental impact and power imbalances.

### 6.1 (Gen)AI bias-driven criminalisation

Discriminative AI algorithms are well-known for embedding several sources of harmful biases and stereotypes against historically marginalised groups within society, and generative AI models are no exception. Research has shown that large language models (LLMs) tend to replicate biases in the training data (78, 79), an issue already prevalent in discriminative algorithms.

For instance, large language models (LLMs) exhibit instances of racial and gender bias when, during in-context impersonation tasks, they describe cars better when asked to impersonate a black person or a male while describing birds better when impersonating a white person or a female (80). Furthermore, an analysis of GPT-2 and GPT-3.5 revealed a propensity to generate masculine-associated pronouns more frequently than feminine-associated ones and show gender-biased association in the context of professions, considering occupations such as Doctor or Engineer as masculine more often than roles like Nurse and Teacher, often regarded as feminine (81). Language-dependent ethnic biases, involving the over-generalised association of an ethnic group to specific attributes, mostly negative, have been found in BERT, where non-toxic comments are incorrectly labelled as toxic when including Middle Eastern country names (82).

Similarly, evidence of religious bias has been found in AI text generators, where the models generate words such as violent, jihad, bomb blasts, terrorism and terrorist at a greater rate in association with the religion Muslim or Islam than with other religions (83, 84).

Biases are also present in the often overlooked dimension of disability; studies have shown that, even when disability is not discussed explicitly, pre-trained language models (PLMs) consistently assign more negative scores to sentences containing words associated with disability compared to those that do not (85). This confirms previous findings indicating that a high percentage of online comments mentioning disabilities on the Jigsaw (86) dataset was labelled as toxic and showed an over-representation of terms related to homelessness, gun violence, and drug addiction, negatively impacting the representation of disability (87).

These systems further suffer from an intersectional bias, where the intersection of different categories of social difference results in new forms of stigmatisation (88).

Those biases are not limited to LLMs but are also visible in Text-to-image (TTI) generative models; for instance, DALL-E 2 has been shown to underrepresent women in stereotypically male-dominated fields while overrepresenting them in stereotypically female-dominated occupations, frequently portraying a higher representation of women than men wearing smiles and tilting their heads downward, particularly in stereotypically female-dominated occupations (89).

Text-to-image (TTI) models’ outputs have also been found to perpetrate identity-based stereotypes, for instance, generating stereotyped images of non-cisgender identities (90) and reproducing Western-centric representations (91, 92), resulting in the reinforcement of whiteness as the ideal standard, the amplification of racial and gender disparities, and the propagation of American-centred narratives (93).

These biased representations can have a profound impact on stakeholders, particularly when integrated into systems used in the forensic domain, where discriminatory outputs and inaccurate formulations have severe implications for all parties.

In fact, forensic psychiatric patients are a population already facing high levels of stigmatisation, as mental illness and criminal history are both commonly associated with social dangerousness, a stereotyped representation widely held in the public perception and permeating society at many levels (94). As a consequence, forensic psychiatric patients are frequently exposed to experiences of rejection and alienation, contributing to a higher risk of internalising negative perceptions held towards them, known as self-stigmatisation (95). Furthermore, these negative stereotypes are used to justify, legitimise and promote legal restrictions and discriminatory practices, such as increased use of coercion (96).

Within the correctional system, pervasive racial stigma intertwines with negative portrayals of forensic psychiatric patients as dangerous and aggressive, contributing to disproportionately high incarceration rates of African Americans (97) and their systemic over-diagnosis with highly stigmatised disorders associated with incompetence, such as psychotic disorders (98). As a result, forensic psychiatric patients face the intersection of multiple stigmatised identities, with damaging effects on self-esteem, depression, therapeutic alliance, and treatment adherence (99).

Within this context, the application of generative AI models in critical tasks that encompass life-altering outcomes, such as risk assessment, sentencing recommendation and treatment and rehabilitation planning, will not only reiterate but significantly magnify existing biases, exacerbating discrimination against forensic psychiatry patients, particularly those from historically marginalised groups, and reinforcing the stigma they experience across multiple levels of society.

For instance, research has shown that, as datasets used by generative AI models expand in scale, there is a noticeable increase in the likelihood of these models classifying Black individuals as ‘criminal’ or ‘suspicious person,’ perpetuating historical and racially biased patterns of criminalisation. Additionally, the deployment of text-to-image (TTI) models in applications like ‘Forensic Sketch AIrtist’ (2022) (100), a forensic sketch program by EagleAI developers utilising DALL-E 2, poses a substantial risk of exacerbating existing racial and gender biases inherent in original witness descriptions while aiming to generate ‘realistic’ sketches of police suspects based on users’ inputs.

In summary, biased AI systems generate significant harm that cannot be overlooked. Generative AI models have the potential to significantly worsen these consequences, exacerbating disproportionate criminalisation of marginalised groups, perpetuating stigmatising attitudes and reinforcing harmful links between mental health and social dangerousness.

### 6.2 Transparency, interpretability, accountability

Understanding and explaining the complexity of generative AI models and their decision-making process to their stakeholders and those affected by their outputs is a challenging task, unveiling significant concerns related to their transparency and interpretability. The opacity of generative models contributes to a lack of accountability, exacerbated by the proprietary nature of the software (79) and by the absence of transparent, ethical oversight during these models’ development, which prioritises hype and profit over ethical and accountable work (101). Additionally, the dominance of industry in AI research, due to its control over crucial resources such as computing power, extensive datasets, and highly skilled researchers, makes it challenging for Academia and the public sector to inquire, monitor, and audit AI models or provide alternative solutions (102), while simultaneously imposing an unfair burden of responsibility on them. The need for transparency and accountability, especially following the widespread adoption of generative AI models, calls for the creation of a regulatory framework tailored to respond to the dynamically changing AI landscape and to address not only the technical aspects but also the broader ethical, societal, and economic implications, promoting their responsible and ethical use (103) while favouring critical enquiries on issues related to responsibility, accountability, and labour exploitation (78).

### 6.3 Data quality, privacy & security

Training large generative AI models (LGAIMs) requires extensive data, often sourced from openly available internet data. This data often contains biased and undesirable content, raising concerns about data quality (104) as well as privacy and security issues. Web-scraped datasets might contain various personally identifiable information about the data subjects, such as their names and email addresses (105); as an example, the metadata scraped by text-to-image (TTI) generative models can include names or other personal information of the authors and the subjects of the media files.

Data privacy and security risks include unauthorised data collection, the risk of re-identification of previously anonymised data, and inadequate data retention practices that could lead to data privacy violations, such as data breaches and unauthorised data sharing.

During training, generative AI models may inadvertently encode and reproduce content containing sensitive data, posing a risk of data leakage. Moreover, even when explicit personal information is absent from the training data, the content generated by generative AI models, when combined with other accessible data, might still lead to the re-identification of individuals or the disclosure of their personal information.

In forensic psychiatry, where access to sensitive data, such as medical, criminal and psychiatric records, is bound to strict legal and ethical regulations, obtaining and using these data without adequate data protection measures violates privacy laws and ethical principles. Consequently, the use of generative AI models in such environments calls for robust regulation to ensure the confidentiality and security of patients’ information, including guidelines for data anonymisation and retention and strategies to prevent data misuse and unauthorised access by external parties (103).

Moreover, if individuals are unjustly detained due to cyberattacks or hacked data, AI companies’ lack of transparency and legal responsibility might leave affected individuals without adequate legal resources (106).

### 6.4 Intellectual property rights & copyright infringements

Although generative AI models gained popularity for their ability to generate novel content, it is crucial to note that the examples used by these models are typically derived from existing human-made works, raising issues of copyright infringement and unauthorised imitation. Large language models (LLMs) are trained on an extensive corpus of data, some of which may have been acquired without proper consent, as the models usually scrape data from the internet, disregarding copyright licenses, plagiarising content, and repurposing proprietary materials without permission.

As a result, it becomes challenging to trace the lineage of the content generated by those models, and due credit is frequently not given to the original creators, potentially exposing users to copyright infringement issues (107, 108) and resulting in legal actions against companies, accused of violating intellectual property rights (109).

### 6.5 Autonomy and informed consent

The widespread adoption of biased and opaque generative AI tools, developed without a robust regulatory framework, which increasingly influence decisions concerning an individual’s psychiatric evaluation, treatment, or legal status, raises concerns about safeguarding individuals’ autonomy and their level of agency over their own information and cases.

AI-driven decision-making tools greatly challenge the principle of respect for the patient’s autonomy, especially in forensic psychiatry applications. In fact, unlike public safety protocols, critical activities such as rehabilitation and forensic mental health evaluations necessitate individuals’ direct and voluntary participation (110).

The lack of transparency surrounding AI algorithms highly compromises the process of obtaining informed consent. Evaluators’ limited understanding of how algorithms generate assessments, including the specific data considered, their respective importance, and the model’s rationale, hinders their ability to effectively communicate this process to the individuals undergoing evaluation (110). This contradicts the fundamental principle of autonomy in medical ethics, which emphasises patients’ control over procedures concerning them, including the use of their data.

Additionally, the incorporation of AI systems in medico-legal decision-making challenges the autonomy of forensic mental health professionals. As an additional factor altering the shared decision-making process between professionals and patients, algorithms undermine clinicians’ perceived authority and impact their judgment. In fact, despite the increasing influence of AI recommendations, in instances where AI judgment conflicts with human judgment, the responsibility to authorise the treatment remains with the professional, who must feel empowered to make autonomous decisions (111).

Furthermore, increased reliance on AI outputs reduces professionals’ use of their own ethical reasoning. Since professionals are responsible for evaluating these outputs, a weakened ethical judgment may impact the criteria used for algorithms assessment (112).

Finally, the application of AI in medico-legal decision-making poses significant challenges to both professionals and patients. If left unregulated, it undermines their authority over crucial decisions that directly influence their lives.

### 6.6 Overreliance

The current debate surrounding ChatGPT and generative AI is dominated by exaggerated and sensationalistic portrayals of their capabilities, resulting in overreliance on their outputs, exacerbating the risk of spreading misinformation and reinforcing biased stereotypes (113).

This overreliance carries profound implications in forensic psychiatry and criminal justice, where outputs of generative AI models increasingly influence clinical assessment and legal decision-making.

For instance, recent news reports have highlighted several instances in which judges and lawyers relied on ChatGPT’s recommendations as a support for decision-making; for instance, a British Court of Appeal judge admitted using ChatGPT to summarise an area of law for a case ruling (114), and a judge in Colombia announced he consulted ChatGPT in preparing a ruling in a children’s medical rights case (115). Similarly, a judge in a Pakistani court used the chatbot to render judgements in a case (116). In another instance, two layers have submitted false evidence generated through ChatGPT in an aviation injury claim (117) - a consequence of the chatbot’s ‘hallucination’, a phenomenon discussed in the following paragraph- which also led to the first major sanction on the use of artificial intelligence within the legal domain.

This growing trend is particularly concerning as it showcases how the widespread availability and ease of access to generative tools contrasts with the lack of awareness of the mechanisms behind their outputs. The situation is further aggravated by the marketing of these models as outstanding and infallible products, often portrayed as possessing human or even superhuman-level reasoning capabilities.

This issue highlights the necessity for AI companies to communicate the genuine potential of their products in a transparent and non-deceptive way, as well as to divulge details about the data used in the models and their analytical processes. Also, it illustrates the need to ensure the digital literacy of legal professionals in critical times of generative AI evidence (115).

### 6.7 Hallucinations, inaccuracy and (mis)facts fabrication

The previously mentioned episode concerning lawyers submitting fake evidence to the court is not an isolated case; in fact, large generative AI models (LGAIMs) have demonstrated tendencies to occasionally generate non-existent and false content, casting doubt on the accuracy of their outputs — a phenomenon called ‘hallucination’.

‘Facts fabrication’ by generative AI models is not limited to the legal context but expands to various settings. For instance, ChatGPT has been shown to produce seemingly plausible but incorrect answers when asked about scientific topics (118) and to fabricate false references for scientific articles (119).

Hallucinations have been associated with disruptions in the language generation process. As large language models (LLMs) generate probabilistic outputs relying on estimations of semantic similarity, when a disruption occurs in this process, it can lead to the integration of false information alongside factual content, raising serious concerns about the trustworthiness of their outputs (120). Hallucinations are primarily associated with LLMs, but they also manifest in models generating video, images and audio; for instance, when Midjourney was tasked with generating images of people enjoying a house party, while the overall scene appeared realistic, a closer look revealed unrealistic elements such as individuals with an excessive number of teeth or hands with more fingers than usual (121).

The fabrication of (false) information risks misleading the users and, especially as a growing number of individuals rely on these tools for guidance and information, the continuous presentation of false information as a factual truth has the potential to distort the perception of reality, acting as a ‘misinformation superspreader’ and resulting in significant harm, especially when inaccurate outputs are used to support forensic decision-making.

### 6.8 GenAI deepfake evidence and the quest for reality

Progress in generative AI models resulted in the production of content that is increasingly challenging to distinguish from human-generated material.

Once evidence generated by generative AI enters the courtroom, it presents significant challenges to all parties. For instance, judges will face the complex task of ruling on an increasing number of disputes over the authenticity of evidence that might be contested as a deepfake.

The judicial system is currently unprepared to handle evidence derived from AI systems, an area in which they possess limited expertise. This compounds the complexity of ruling on digital evidence and creates a demand for technical experts knowledgeable in generative AI and deepfake technologies, further increasing costs and time duration of legal proceedings (122).

Moreover, the growing probability of encountering AI-generated evidence in courtrooms is likely to instil a sense of doubt and scepticism amongst judges, juries and the general public, fostering an environment where all parties are inclined to consider the possibility that their counterparts have submitted AI-generated evidence - a phenomenon also referred to as “the deepfake defence” (123), which ultimately pollutes the decision-making processes.

This phenomenon will create an environment characterised by an overarching sense of distrust, in which parties can weaponise scepticism and doubts to advance their own agendas, a concept also known as the “liar’s dividend” (124).

Additionally, the advancement of tools for detecting AI-generated content raises questions about which content will likely be more targeted and the potential legal consequences of identifying AI-generated evidence. At present, AI-generated content detectors are insufficiently accurate and show notable inconsistency in categorising content as either AI-generated or human-written (125).

In forensic psychiatry, where research suggests that juries and judges tend to misinterpret scientific evidence in court, for instance overestimating the reliability of neuroscientific evidence (126), leading to miscarriages of justice (127), the potential introduction of genAI-fabricated evidence introduces the risk of wrongful convictions grounded in maliciously AI-generated scientific evidence.

In summary, the rise of generative AI introduces a concerning scepticism that could disrupt decision-making at an individual and societal level, underscoring the growing need to preserve our rights to reality in this evolving era of AI.

### 6.9 Environmental impact and sustainability

As we delve into discussing AI’s impact and ethical development, it is imperative to mention that the impressive capabilities of generative AI models come at a hidden and frequently overlooked environmental cost. In fact, alongside the usage and continuous development of generative AI models, the computational power required to train them and maintain their physical infrastructure grows together with their carbon emissions, raising concerns from a climate policy perspective (128–130).

Although these tools are currently in the early stages of gaining mainstream adoption, it is reasonable to anticipate that their environmental costs will grow significantly in the coming years. Consequently, it is crucial to develop metrics to evaluate the environmental impact of AI development to identify strategies to mitigate it (131).

### 6.10 Power, homogeneity and ‘bias-in-the-loop’

A discussion about AI Ethics must encompass an analysis of power dynamics; understanding the positionality of the stakeholders and their respective levels of influence is, in fact, crucial for gaining insight into the potential hazards of AI.

Algorithmic bias is a symptom of a broader issue about power imbalances and historical inequities that influence AI technologies’ creation, deployment, and objectives, starting from how data is collected and managed, including the authority in deciding which aspects are measured and included in the datasets (132). Technology is not neutral; AI solutions are value-laden and are “specified by developers and configured by users with desired outcomes in mind that privilege some values and interests over others” (133). Nowadays, there is a substantial disparity in the AI domain between the Global North and the Global South, wherein the latter is often subjected to exploitation for low-cost or unpaid labour, meanwhile the main benefits and advancements are concentrated in the Global North. As a result, individuals from the Global North gain early access to cutting-edge generative AI tools, while marginalised groups are left behind, causing issues of unequal access and exacerbating the existing disparities in the technological landscape.

Moreover, since companies employ user input to train their models, such as OpenAI, which may use content entered by users in ChatGPT to improve the model’s performance (134), this process could introduce an additional ‘bias-in-the-loop’, where countries and individuals who get to access and use generative AI models will further control and shape their outputs through their inputs and queries, thereby intensifying digital disparities.

The emergence of generative AI has widened several layers of digital divides, holding significant implications for offline outcomes and amplifying digital inequalities. As a consequence, individuals lacking access to extensive data resources face vulnerability when comprehending the data and methodologies employed in decisions that impact them. The problematic nature of algorithmic decision-making, marked by an asymmetry in knowledge and decision-making authority, significantly exacerbates this vulnerability (135). The issue is intensified by significant power imbalances in the criminal justice system resulting from detention under mental health legislation, where forensic psychiatric patients often have limited access to technology, worsening disparities in access to information and communication resources.

Additionally, the widespread use of generative AI raises concerns about the diffusion of increasingly uniform outputs generated by AI models trained on a limited range of references. This homogenisation extends not only to language, communication styles, and public discourse but also to economic power and information, consolidating economic influence within a few organisations governing AI systems, fostering economic homogeneity and inequality.

To establish an ethical and responsible AI framework, it is imperative to integrate diverse perspectives and voices at every stage of the AI process, from dataset creation and curation to model development and utilisation. This imperative is inseparable from efforts to renegotiate and redistribute power. Without initiatives to rebalance power dynamics, the prospects for democratising AI and ensuring its responsible use remain elusive, especially within the biased criminal justice system.

## 7 Conclusions

The rapid advancements of technology and the widespread use of generative artificial intelligence in several fields require society to match the pace of these developments. Currently, we are falling short in this regard, allowing AI’s outcomes to impact our lives prior to undergoing comprehensive investigation and regulation.

This article discusses the impact of generative AI in forensic psychiatry and criminal justice, analysing current and prospective applications while drawing comparisons with the previously dominant paradigm of discriminative AI.

This comparative exploration reveals the convergence of both past and emerging challenges. First, it becomes evident that generative AI not only holds the potential to revolutionise traditional discriminative tasks, for instance, by leveraging its enhanced analytical capabilities to enhance risk assessment and diagnostic support, but also to open avenues to previously overlooked applications, like AI-powered simulations for training and educational purposes.

When exploring the ethical and legal issues, the analysis shows that generative AI models not only inherit the prevailing challenges present in discriminative AI algorithms, such as biased and stereotyped outputs, lack of transparency, and data privacy issues, but also amplify their impact, due to heightened computational capabilities and increased accessibility and ease of use. Furthermore, generative AI models introduce novel and unique challenges, such as hallucinations and facts fabrication, progressive homogenisation of content, and concerns about data quality and intellectual property rights. Specifically, within forensic psychiatry, some of the most concerning aspects include the spread of misinformation and the reinforcement of discriminatory and criminalising narratives and stereotypes. This unfolds as a result of the increasing overreliance on AI-generated outputs used by judges, legal experts, and mental health practitioners in their decision-making processes. The situation becomes particularly problematic if biased outputs are employed for training and educational purposes, as they could have a negative impact on the perspectives and knowledge of future forensic mental health professionals.

In fact, large generative AI models carry the potential to strengthen the negative association between mental health and criminal history; as a consequence, there will be an increased risk of criminalisation of forensic psychiatry patients, especially those belonging to historically oppressed groups, alongside with enhanced profiling, mass surveillance and unfair allocation of resources and treatment assignments.

While unregulated industry controls resources and power, institutions need to provide society with the necessary tools to investigate and hold those systems accountable. Continuous discussions and collaborations among stakeholders, including forensic psychiatrists, AI developers, legal experts, and ethicists, are essential to navigating these complex issues, while considering the diversity in forensic psychiatry practices shaped by differences in healthcare and legal systems among different countries. Additionally, maintaining an ongoing dialogue with affected communities, who often lack representation in these discussions, and involving them in the process, is crucial.

Lastly, as algorithms and their decision-making are a reflection of society, we need to work on shifting from a surveillance-based approach to one focused on tackling the root causes of criminalisation and inequality, emphasising the safeguard of mental health and rehabilitation over criminalisation and profiling.

## Author contributions

LT: Conceptualization, Writing – original draft, Writing – review & editing.
